# Design and Biofunctionalization of Cloud Sponge-Inspired Scaffolds for Enhanced Bone Cell Performance

**DOI:** 10.1021/acsabm.4c01065

**Published:** 2024-11-16

**Authors:** Philipp Zimmermann, Peter Schulze, Annette G. Beck-Sickinger, Yuliya Khrunyk

**Affiliations:** † Engineering Faculty, Leipzig University of Applied Sciences (HTWK), Karl Liebknecht Str. 134, D-04277 Leipzig, Germany; ‡ Institute of Biochemistry, Faculty of Life Sciences, Leipzig University, Brüderstrs. 34, D-04103 Leipzig, Germany

**Keywords:** mussel peptide MP, cyclic RGD, porosity, sponge-inspired scaffolds, Clear Resin, bone cells

## Abstract

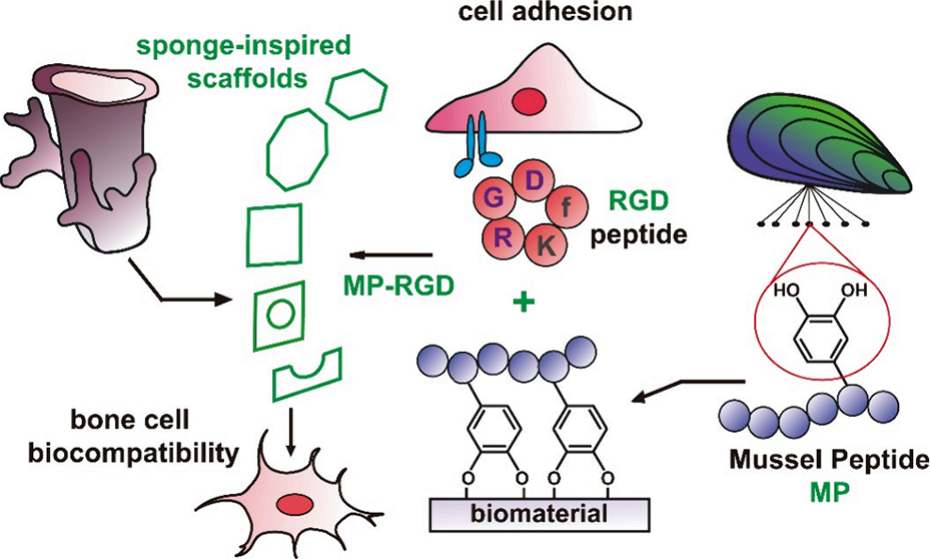

With the increasing age of our population, which is linked to a higher incidence of musculoskeletal diseases, there is a massive clinical need for bone implants. Porous scaffolds, usually offering a lower stiffness and allowing for the ingrowth of blood vessels and nerves, serve as an attractive alternative to conventional implants. Natural porous skeletons from marine sponges represent an array of evolutionarily optimized patterns, inspiring the design of biomaterials. In this study, cloud sponge-inspired scaffolds were designed and printed from a photocurable polymer, Clear Resin. These scaffolds were biofunctionalized by mussel-derived peptide MP-RGD, a recently developed peptide that contains a cyclic, bioactive RGD cell adhesion motif and catechol moieties, which provide the anchoring of the peptide to the surface. In *in vitro* cell culture assays with bone cells, significantly higher biocompatibility of three scaffolds, **i.e.**, square, octagon, and hexagon cubes, in comparison to hollow and sphere inside cubes was shown. The performance of the cells regarding signaling was further enhanced by applying an MP-RGD coating. Consequently, these data demonstrate that both the structure of the scaffold and the coating contribute to the biocompatibility of the material. Three out of five MP-RGD-coated sponge-inspired scaffolds displayed superior biochemical properties and might guide material design for improved bone implants.

## 1 Introduction

Today the treatment of larger bone fractures (segmental bone defects) is still one of the main challenges in implantology. The use of autologous bone grafts (based on the harvesting of nonessential bone) can lead to donor site morbidity, infection, and geometric mismatch between the defect site and the harvested bone.^1^ On the other hand, scaffolds to be used as bone implants must be biocompatible with the natural bone. In implantology, implant failure is often associated with a mismatch in stiffness between the implanted material and human bone.^2,3^ Indeed, the elasticity (Young’s) modulus *E* of human trabecular bone and cortical bone (in their hydrated state) varies from 3 to 18 GPa^4^ and from 18 to 22 GPa,^5^ respectively, which is significantly lower than *E* values reported for metallic materials.^6−9^ In implants with porous designs, however, the Young’s modulus can be reduced depending on pore parameters, *i.e.*, porosity, pore size, and pore distribution.^10,11^ Furthermore, the scaffold’s geometry, including pore size and pore interconnectivity, is linked to nutrient transport and extracellular matrix formation affecting cell attachment and proliferation.^12−15^

The design and manufacturing of porous scaffolds with complex geometries that could also be personalized to fit the surface area of a specific patient became possible following the advances in 3D printing. Additive manufacturing (AM) technology allows us to manufacture scaffolds with controllable “single unit” shape, pore size, surface area, material volume, and porosity. In the search for the designs of novel, porous 3D scaffolds, the use of the biomimetics approach offering a plethora of natural, evolutionarily carved structures as an inspiration has been gaining growing attention. In particular, glass sponges (Hexactinellida) offer an abundance of scaffolds that have undergone over 500 MYA of evolutionary selection (from the Late Proterozoic period), optimizing their strength, flexibility, and low-weight properties.^16,17^ The scaffold of cloud sponge *Aphrocallistes vastus*, a member species of this family, is characterized by a porous honeycomb-like structure composed mostly of hexagons, a pattern that can be widely observed in other nonrelated species, *i.e.*, the wing scales of *Parides sesostris* and *Teinopalpus imperialis* butterflies,^18^ tissues of giant reed *Arundo donax*,^19^*Drosophila* eyes,^20^ etc. The presence of the honeycomb structure in marine sponges, however, indicates that this pattern already existed at the dawn of multicellular evolution. In the search of scaffold designs facilitating initial cell adhesion and proliferation, a variety of 3D permeable sponge-inspired scaffolds can be constructed depending on the geometry and size of structural units, their relative density, and the composition of the solid material they are made from.^21^

The biofunctionalization of printed 3D scaffolds with bioactive peptides, *i.e.*, RGD peptides, can further enhance their biocompatibility, in particular regarding the response of bone cells.^22−27^ Such RGD-containing peptides (including more stable cyclic RGD peptides) can be immobilized on the surface using a mussel-derived peptide (MP), the sequence of which has been inspired by the adhesive proteins of the blue mussel (*Mytilus edulis*).^23,28−30^ While functioning as an anchor owing to the presence of l-3,4-dihydroxyphenylalanine (DOPA), MP can be further “decorated” with a cyclic RGD by an orthogonal click reaction,^31^ which provides an opportunity to combine cell adhesion motif and “biological glue” properties in a single molecule.

In this study, we aimed (i) to design and print a set of 3D sponge-inspired porous scaffolds, (ii) biofunctionalize them with RGD-containing biomimetic peptides, and (iii) characterize their biocompatibility in *in vitro* cell culture assays employing bone cells.

## 2 Results

### 2.1 SEM and micro-CT Analysis of the *A. vastus* Scaffold

**Figure 1:**
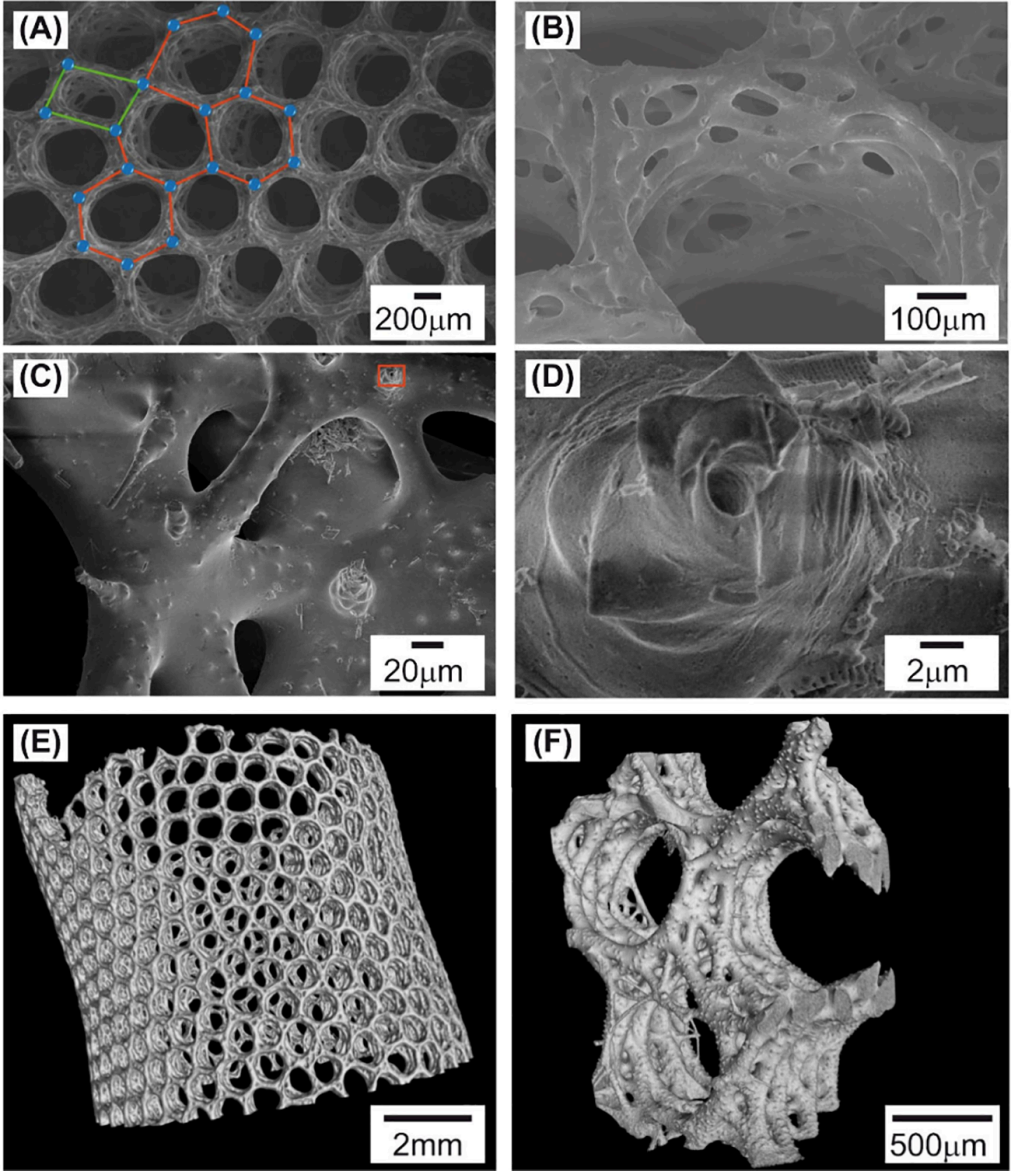
(A–D) SEM analysis and (E, F) micro-CT scanning of the *A. vastus* scaffold. (A) Honeycomb pattern with hexagon-shaped cells, the edges and nodes of which are highlighted by red lines and blue circles. Geometrical defects (*i.e.*, tetragon-shaped cell) were also visible, as marked by green lines. (B) An image of tubular cavities (diarhyses) formed by fused spicules. (C) A closer view of spicules forming a diarhyse. (D) An enlarged view showing an axial canal (marked as a red rectangle in (C)). (E) A whole tubular-shaped scaffold. (F) A close image of diarhyse cavities.

The scaffold of *A. vastus* has the shape of a tube, the lateral walls of which show honeycomb-like structures formed by tubular canals (diarhyses). Scanning electron microscopy (SEM) analysis indicated that these structures resembled a mesh made of “edges” and “nodes”, which formed hexagon pores (Figure 1A). Not only did the shape and size of the hexagons differ but also some hexagons degenerated into other geometric shapes, *i.e.*, tetragons. A closer view of a diarhyse (Figure 1B,C) displays fused spicules forming triangle-like patterns. The surface of the spicules does not look smooth but instead bubbled, perhaps due to some inclusions. Axial canals were observed in not fused and broken spicules (Figure 1D).

The scanning of the *A. vastus* scaffold using microcomputer tomography (micro-CT) allowed us to visualize a 3D structure of a complete scaffold or its large parts (Figure 1E,F) showing the prevalence of hexamer-shaped cells.

### 2.2 3D Reconstruction of the *A. vastus* Scaffold

**Figure 2:**
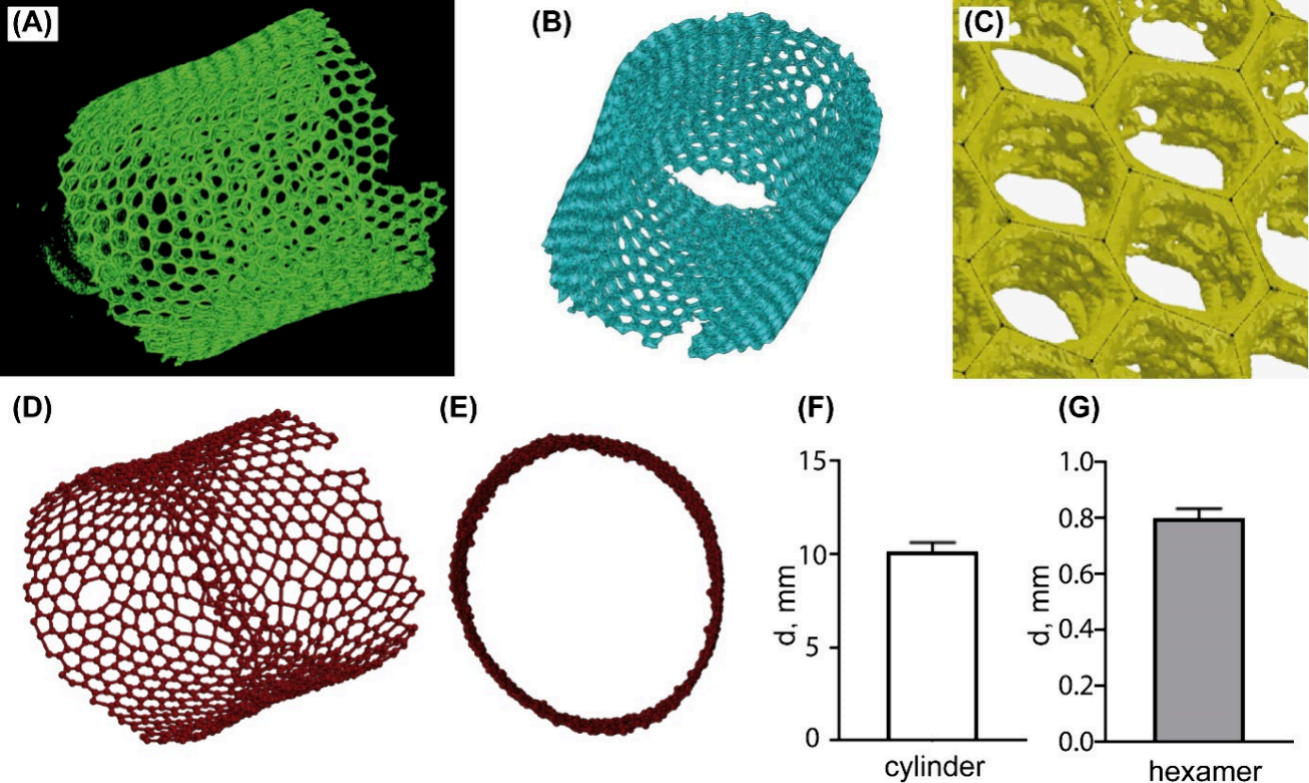
Steps of the 3D reconstruction of the *A. vastus* scaffold. (A) The analysis of DICOM images obtained using microcomputer tomography in InVesalius 3.1. (B) Final STL file generated in InVesalius 3.1. (C) 3D sketch in SolidWorks software. (D) CAD 3D model fully replicating the shape and geometry of original *A. vastus* scaffold. Isometric view. (E) Top view. Morphometrical analysis of *A. vastus* scaffold for (F) the diameter of a scaffold cylinder (*n* = 20) and (G) a circle inscribed in a hexamer (*n* = 872) as mean ± SD.

The results of SEM and micro-CT indicate that the *A. vastus* honeycomb scaffold does not consist of a distinct repetitive element. In order to evaluate the quantity and peculiarities of such “honeycomb monomers”, a 3D model of the *A. vastus* honeycomb structure has been constructed (Figure 2A–E).

Following a series of measurements, mean values of the tube diameter (10.131 mm) and the diameter of circle inscribed in a hexamer (0.800 mm) were obtained (Figure 2F,G). The analysis of all honeycomb monomers indicated that hexagons account for 80% of all cells. Also, 17% of cells are composed by pentagons. A small ratio of other geometric cells was detected, *i.e.*, heptagons (2.5%) and octagons and tetragons (less than 1%). Out of 1090 nodes analyzed, 1055 nodes were connected with three cell edges and 35 with four cell edges.

### 2.3 Design and Characterization of Sponge-Inspired 3D Porous Scaffolds

**Figure 3:**
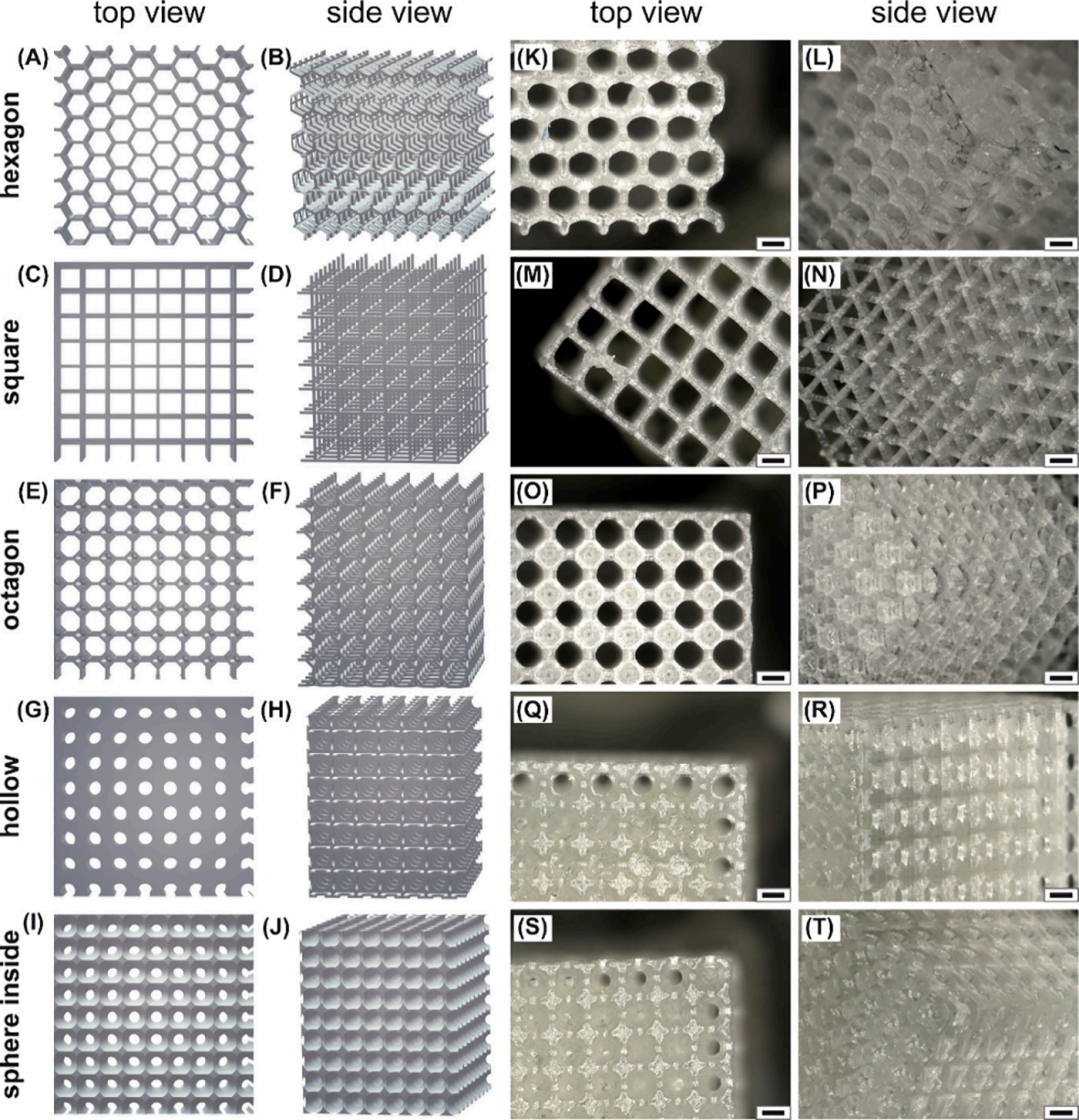
(A–J) STL images of designed scaffolds having different geometrical architectures. (K–T) The visualization of scaffolds printed from Clear Resin, *i.e.*, (K, L) hexagon, (M, N) square, (O, P) octagon, (Q, R) hollow, and (S, T) sphere inside cubes. The microscopy was carried out using a Keyence digital microscope VHX-7000 (Osaka, Japan) with a VH-Z20R objective lens. Scale bar: 500 μm.

A set of porous 3D scaffolds having the shape of a cube, *i.e.*, hexagon, square, octagon, hollow, and sphere inside cubes, have been designed (Figure 3A–J) and printed from the commercial polymer Clear Resin (CR) (Figure 3K–T). Additionally, control nonporous cubes of the same size were printed from CR.

**Figure 4:**
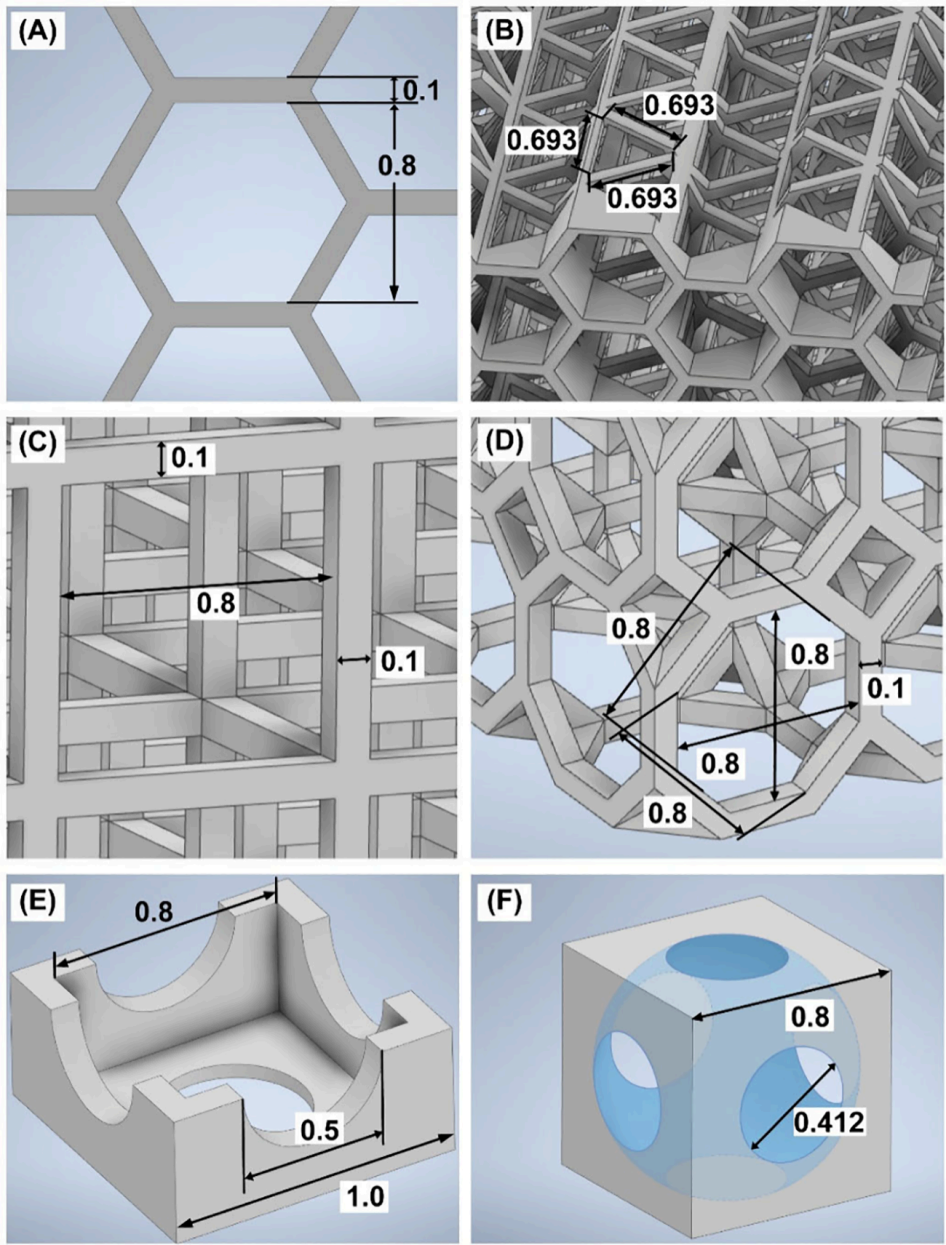
Structural properties of porous scaffolds (in mm): hexagon cube formed by (A) hexagons and (B) triangles, (C) square cube, (D) octagon cube, (E) monomers of the hollow cube, and (F) sphere inside cube.

This polymer proved to be suitable to maintain the printing of structures, the design of which was inspired by the dimensions of cloud sponge honeycombs. More detailed information on the design of printed scaffolds is provided by Table S1 and Figure 4.

Except for the hexagon scaffold (the structure of which replicates cloud sponge elements, Figure 4A,B), the square, octagon, hollow, and sphere inside scaffolds consist of cube-shaped unit cells that can be arranged in all three dimensions to scale up to the actual size of the sample. The size of their structure parameters is comparable to that of the hexagon cube (Figure 4). Square and octagon cubes are made up of square- and octagon-like elements (Figure 4C,D). In a hollow cube, the smaller cube is cut out of the bigger one with holes added on all the sides (Figure 4E), while in the sphere inside cube the sphere is cut out of the unit cell cube, with the sphere having a diameter larger than the cube’s side length, also forming holes on all the faces (Figure 4F).

### 2.4 Biocompatibility of Clear Resin Scaffolds

**Figure 5:**
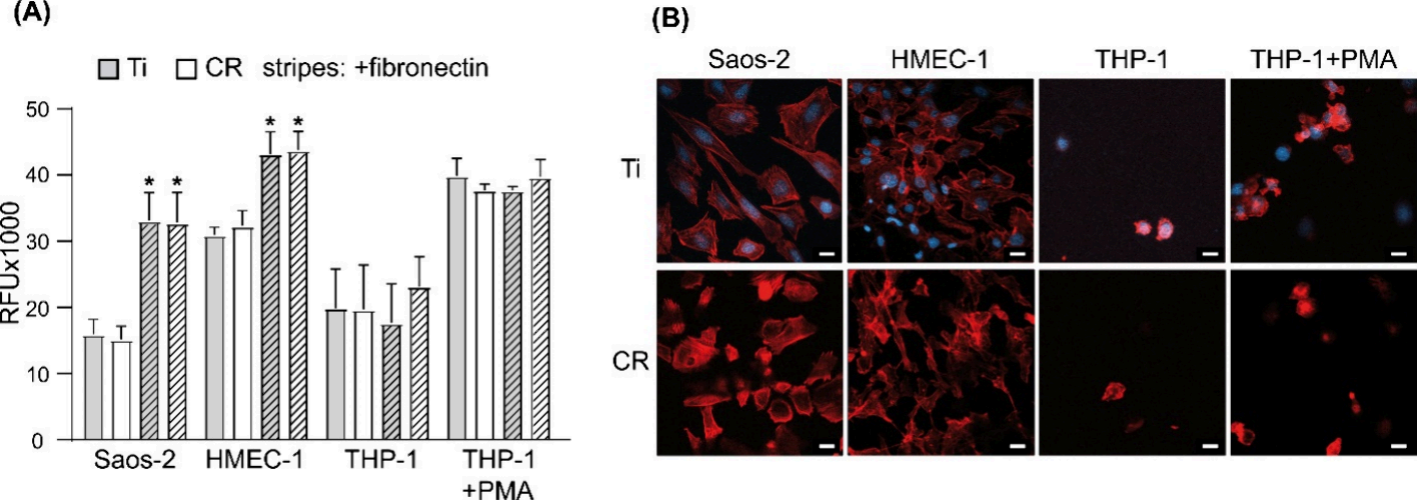
Biocompatibility of scaffolds made out of CR. (A) The results of resazurin reduction in cell cultures: Saos-2, HMEC-1, and THP-1 (without and with phorbol 12-myristate 13-acetate (PMA)) at 24 h after seeding, indicating mitochondrial activity. Tested scaffolds were cultured on titanium and CR discs without and with a fibronectin coating. The measurements were taken at 120 min following incubation with resazurin; RFU: relative fluorescence units; * for *p* ≤ 0.05, data represent mean ± SEM, *n* = 3. (B) Cell adhesion on titanium and CR discs. Saos-2, HMEC-1, and THP-1 (without and with PMA) were cultured on the analyzed scaffolds for 24 h. Cell actin and nuclei were stained with TRITC-Phalloidin (red) and Hoechst 33342 (blue), respectively. On CR, nuclei are not visualized due to the autofluorescence of CR in the blue channel. Sb: 20 μm.

After 24 h of cultivation, the mitochondrial activities of cells seeded on tested substrates, *i.e.*, pure titanium (Ti) and CR discs, uncoated and with a fibronectin coating, were analyzed by the conversion of resazurin to resorufin (Figure 5A). For all cells tested (Saos-2, HMEC-1, and THP-1), resazurin conversion did not differ significantly between Ti and CR, although the biocompatibility of fibronectin-coated substrates (both made of titanium and CR) was significantly higher in comparison to that of uncoated discs. The staining of the cells’ cytoskeleton did not show the differences regarding cell attachment to tested substrates (Ti and CR), indicating that the biocompatibility of CR is comparable to that of Ti (Figure 5B).

### 2.5 Biofunctionalization of Clear Resin with MP-RGD Peptide

**Figure 6:**
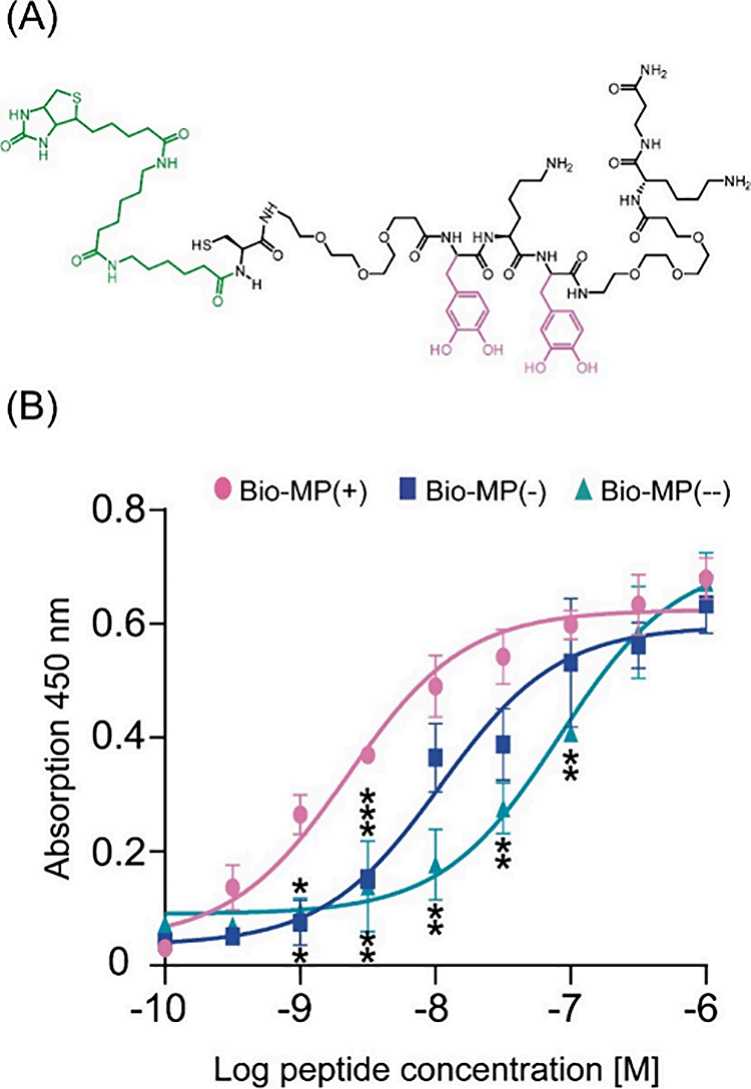
Surface binding properties of mussel-derived peptides to Clear Resin. (A) The chemical structure of Bio-MP(+), equipped with aminohexanoic acid spacers and a biotin tag (green color). In the case of Bio-MP(−) and Bio-MP(--) serving as negative controls, two DOPA (pink color) were substituted by tyrosine (Bio-MP(−)) and phenylalanine (Bio-MP(--)). The chemical structures of Bio-MP(−) and Bio-MP(--) are shown in Figure S1. (B) An elevated binding affinity of Bio-MP(+) to CR in comparison to Bio-MP(−) and Bio-MP(--) was shown in an ELISA-like assay; *p ≤ 0.05, **p ≤ 0.01, ***p ≤ 0.001 above Bio-MP(−) and below Bio-MP(--) value points show significance in comparison to Bio-MP(+) values; *n* = 3.

The biocompatibility of materials to be used for implant manufacturing can be enhanced by coating with bioactive peptides. A cyclic RGD peptide that has a high affinity and selectivity for integrins expressed on osteoblasts can be attached to the material surface employing a mussel-inspired approach, *i.e.*, being linked to a mussel peptide MP. Indeed, due to the presence of l-3,4-dihydroxyphenylalanine (DOPA), MP serves as a tool for the biofunctionalization of scaffolds with specific peptides. In order to test the binding capacity of MP to CR, three biotin-tagged peptide derivatives (Bio-MP) were synthesized (Figure 6A, Figure S1 A,B). Whereas Bio-MP(+) contains two DOPA residues, these have been replaced by Tyr (in Bio-MP(−)) or Phe (in Bio-MP(--)). The identity and purity of the peptides are shown in Figures S2A–C and S3A–C. Discs made of CR were incubated with Bio-MP(+), Bio-MP(−), and Bio-MP(--) in the range of concentrations from 0.1 nM to 1 μM. The detection of biotinylated peptides bound to CR surfaces was conducted using an ELISA-like assay based on the biotin–streptavidin interaction. In an ELISA-like assay, a significantly higher affinity of Bio-MP(+) to CR (in comparison to Bio-MP(−) and Bio-MP(--)) was shown for the concentrations above 10^–9^ M (Figure 6B). In particular, at the concentration of 10^–8.5^ M, 2.4- and 2.7-fold higher affinities with respect to those of Bio-MP(−) and Bio-MP(--) were detected, pointing to an essential role of catechol units in the binding of MP to CR surfaces.

Cyclic RGD has been ligated to MP by means of a Diels–Alder reaction with inverse electron demand (Figures S1C–E, S2D, and S3D): resin bound MP-diene (Figure S1D), modified at the Lys side chain, was incubated with an aqueous solution of c[RGDfK(dienophile)] (Figure S1C) to yield the MP-RGD (Figure S1E) conjugate, which was further used for the coating of printed 3D scaffolds.

### 2.6 Cell Response to MP-RGD-Functionalized Scaffolds

**Figure 7:**
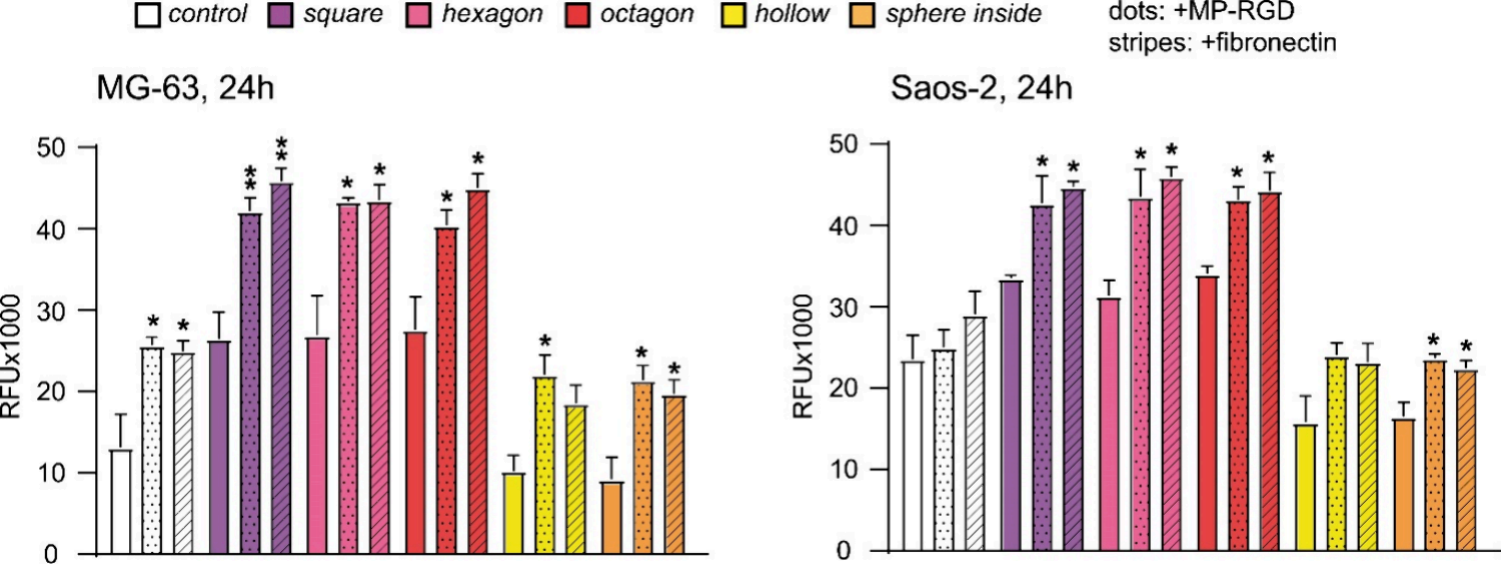
Resazurin reduction in MG-63 (left) and Saos-2 (right) cell cultures 24 h after seeding, indicating mitochondrial activity. Cells were cultured on uncoated scaffolds and coated scaffolds (MP-RGD, 1 μM, dots; fibronectin, 25 μg/mL, stripes). The measurements were taken at 120 min following incubation with resazurin; RFU: relative fluorescence units; *p ≤ 0.05, **p ≤ 0.01, significance is shown in comparison to uncoated scaffolds, data represent mean ± SEM, *n* = 3.

The biocompatibility of sponge-inspired 3D scaffolds (control, square, hexagon, octagon, hollow, and sphere inside cubes) was tested on two osteoblast-like cell lines, *i.e.*, MG-63 and Saos-2. Along with uncoated scaffolds, two types of coatings have been analyzed, *i.e.*, the coating with MP-RGD (1 μM) and fibronectin (25 μg/mL); the latter served as a positive control. The mitochondrial activity of bone cells seeded on tested scaffolds was analyzed after 24 h (Figure 7) and 72 h of cultivation (Figure S4).

After 24 h of cultivation, resazurin reduction in MG-63 and Saos-2 seeded on square, hexagon, and octagon scaffolds was about two times higher in comparison to that on hollow and sphere inside cubes. These values were even further elevated when the coating with MP-RGD was applied. For both bone cell lines tested, the mitochondrial activity of cells seeded onto square, hexagon, and octagon cubes was significantly enhanced in comparison to that on uncoated scaffolds. Their coating with fibronectin also led to a significant inrease in mitochondrial activity, which was comparable with the effect of MP-RGD. Though hollow and sphere inside cubes showed much lower resazurin reduction values, their performance was also improved by the coatings with bioactive peptides, *i.e.*, for hollow cubes, a significant growth in mitochondrial activity was observed in MG-63 cells, while for sphere inside cubes the fluorescence levels significantly increased in both MG-63 and Saos-2 cells. The biocompatibility of control cubes was higher than that of hollow and sphere inside scaffolds but lower in comparison with square, hexagon, and octagon ones.

At 72 h of cell culture, no significant differences between coated and uncoated scaffolds were observed (see Figure S4), although the tendency of square, octagon, and hexagon scaffolds to show higher values of resazurin redaction remained.

**Figure 8:**
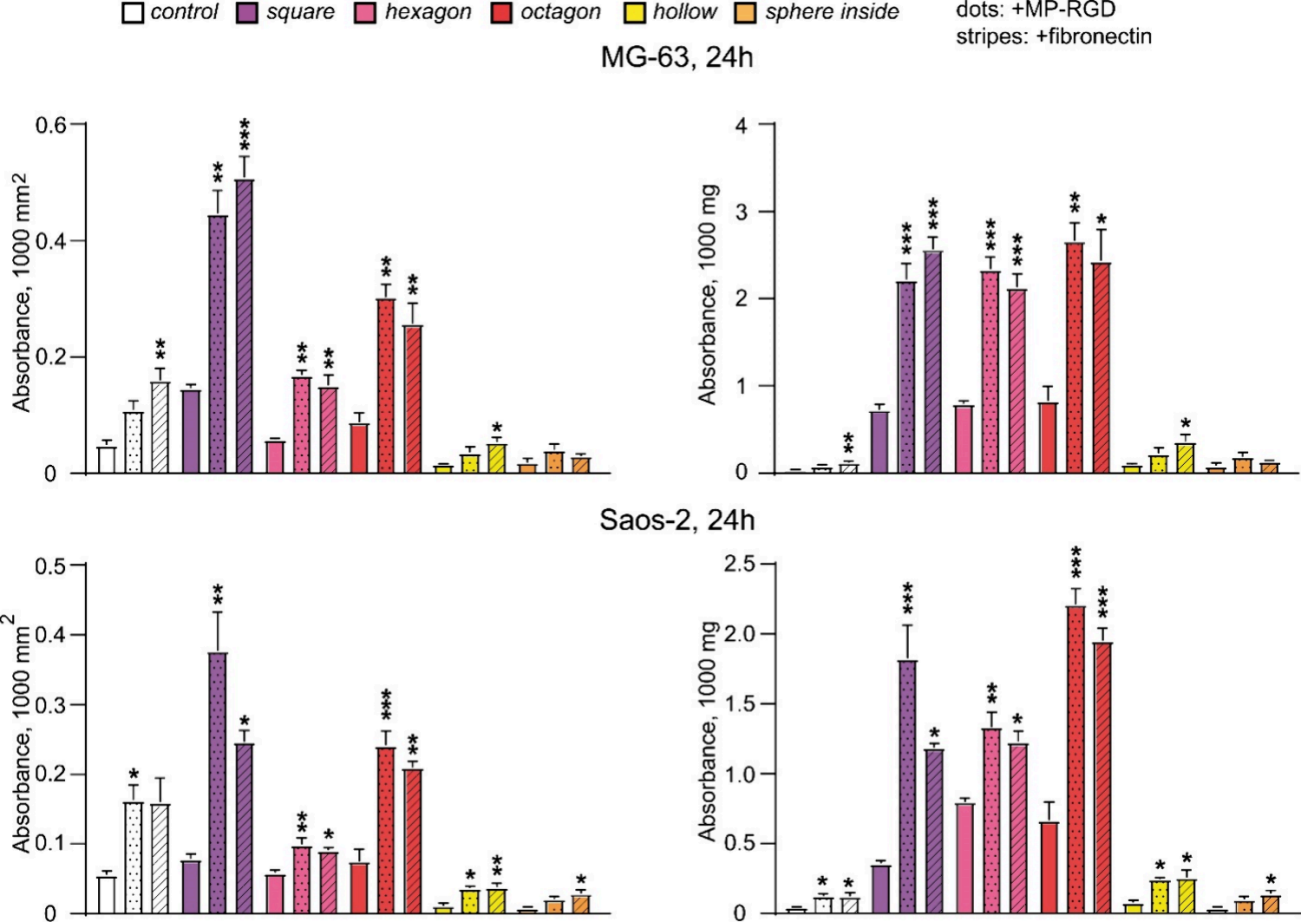
Sulforhodamine B (SRB) assay detecting scaffold biocompatibility based on cellular protein content. SRB absorbance is shown for tested scaffolds seeded with MG-63 and Saos-2 cells for a 24 h cell culture, normalized to the scaffold’s surface area (1000 mm^2^, left) or mass (1000 mg, right). Cells were cultured on uncoated scaffolds and coated scaffolds (MP-RGD, 1 μM, dots; fibronectin, 25 μg/mL, stripes). *p ≤ 0.05, **p ≤ 0.01, and ***p ≤ 0.001, significance is shown in comparison to uncoated scaffolds, data represent mean ± SEM, *n* = 3.

These data were further confirmed by cellular protein content analysis (Figure 8), which relies on the property of sulforhodamine B (SRB) to bind stoichiometrically to proteins. Detected SRB absorbance values were normalized to the surface area and the mass of the scaffolds. The obtained data pointed to a significant increase in cell number for MP-RGD and fibronectin-coated square, octagon, and hexagon cubes seeded with both tested cell lines after both types of normalization. Along with these cubes, the effect of the MP-RGD coating was also observed in control and hollow cubes seeded with Saos-2 cells following both types of normalization. Alike to the resazurin conversion assay, the values of SRB absorbance pointed to a better performance of square, octagon, and hexagon cubes in comparison to hollow and sphere inside cubes, while the coating with MP-RGD amplified these values by about two times. Following both types of normalization, the impact of MP-RGD did not differ from the results shown for fibronectin. Similar to SRB absorbance results, with normalizing the values on resazurin conversion (24 h post-seeding) to the cubes’ surface area and mass (Figure S5), a positive effect of the tested coatings was shown. These data were also confirmed by the visualization of cell viability and the cell cytoskeleton pointing to poorer cell attachment onto uncoated CR in comparison to CR biofunctionalized with MP-RGD and fibronectin (Figure S6).

In line with the data shown by resazurin conversion for cells at 72 h cell culture, the protein content analysis of scaffolds at the same time point showed less obvious differences between scaffolds and insignificant differences between coated and uncoated scaffolds (Figure S7).

## 3 Discussion

Today, biomimetics has sparked a new wave of interest in the design of materials, particularly for biomedical use. Indeed, bioinspired porous scaffolds enabling cell ingrowth and vascularization can be used to repair large segmental bone defects.

By employing micro-CT and software packages (InVesalius 3.1, SolidWorks), a direct replication of a cloud sponge scaffold including naturally occurring irregularities was obtained. This has enabled us to analyze the sponge scaffold structure regarding the geometry of its honeycomb cells, define cells’ dimensions, and design sponge-inspired cubic scaffolds containing unified repetitive elements optimized for additive manufacturing. Obviously there are various technologies that could be used to obtain 3D porous scaffolds (gas foaming, solvent casting, particulate leaching, freeze-drying, phase separation, and electrospinning); however, they have a geometric freedom limitation and hence cannot be applied to manufacture scaffolds with precisely controlled scaffold morphology, including pore geometric parameters. On the other hand, bioinspired complex structures with controlled precise geometric features can be manufactured using AM.

In this research, we have designed and printed the following CAD 3D models of scaffolds: (i) hexagon cube, (ii) octagon cube, (iii) square cube, (iv) hollow cube, and (v) sphere inside cube, along with (vi) control cube, aiming to test their biological compatibility toward bone cells. All the porous scaffolds contained a structural element of about 800 μm, matching the diameter of the cloud sponge honeycomb. The parameters of the cubes’ pores matched the size of trabecular bone pores, *i.e.*, 500–1000 μm.^32,33^ Similar to cloud sponge scaffolds, hexagon cubes were composed of hexagon and triangle elements replicating a sponge structure but in a much more patterned manner. Other porous cubes contained structural elements of comparable dimensions.

All of the cubes were printed from a commercial photopolymer resin Clear Resin (CR) (Formlabs, USA). This resin, along with High Temp and Black resins (Formlabs), was reported to be capable of printing complex structures, *i.e.*, containing slots from 0.4 to 1.2 mm, which apparently was not possible with Dental SG, Dental LT, and Flexible resins (Formlabs).^34^ The exact composition of CR remains proprietary, though the MSDS sheets point to the presence of 55–75% w/w urethane dimethacrylate, 15–25% w/w methacrylate monomers, and <0.9% w/w diphenyl(2,4,6-trimethylbenzoyl)phosphine oxide.^35^ Indeed, containing acryalate- or methacrylate-based monomers and oligomers, commercial resins, *i.e.*, CR, Black Resin, Dental SG, Dental LT, BioMed (Formlabs), and BV007a (MiiCraft), are polymerized under UV or visible light exposure by means of photo initiator(s) and possibly photosensitizers, as well as additives, *i.e.*, stabilizers, fillers, plasticizers, dyes, photoabsorbers, and other compounds improving the quality of printed products.^36−39^

Knowledge on the biocompatibility of such resins is quite scarce, although it was shown that uncured commercial resins are cytotoxic.^40^ In our research, the scaffolds printed from CR were cured and additionally postwashed by sonication in 70% isopropyl alcohol for 30 min, as was proved to be efficient for the cleaning of metallic scaffolds^9^ and reported to significantly improve the viability of cells in the literature, *i.e.*, HL-1 cells^40^ and mice splenocytes^41^ cultured on CR substrates. In addition, the scaffolds were postcured under UV irradiation for 4 h, which is supposed to activate a further cross-linking of the polymer.^42−44^ The biocompatibility of CR, evaluated by mitochondrial activity and microscopy, was comparable to that of pure titanium (medical grade), did not induce the adhesion of macrophage THP-1 cells, and could be improved by the fibronectin coating. These findings are comparable to the studies^41^ showing that the viability of primary murine splenocytes cultured on CR (along with BioMed Resin) was comparable to that of the control (well plate control) in contrast to BV007a Resin (Formlabs) showing cytotoxicity.

The comparative analysis of the cubes’ performance in *in vitro* studies pointed to significant differences regarding cell responses: hexagon, octagon, and square cubes performed better in bone cells culture assays, as was shown by the reduction of resazurin and protein content. These cubes, in contrast to hollow and sphere inside cubes, are characterized by a higher porosity according to CAD, *i.e.*, 86% (hexagon), 96% (square), and 94% (octagon) vs 79% (hollow) and 72% (sphere inside), and according to the experimental data on the cubes’ porosity, which was established by the method of liquid displacement with absolute ethanol, *i.e.*, 79.8% (hexagon), 62.4% (square), and 72.1% (octagon) vs 48.6% (hollow) and 34.1% (sphere inside). Presumably, the porosity of about 70% or higher (shown for hexagon, square and octagon cubes) allows a good permeability of cell culture media, nutrients and growth factors being beneficial for tissue engineering scaffolds.^45^

In order to further optimize the biocompatibility of tested scaffolds, cyclic RGD peptide, carrying an arginine-glycine-aspartic acid (RGD) sequence and known to foster cell adhesion and the regeneration of tissue, was used.^23,46−53^ In particular, a positive effect of RGD-coated materials on osteogenesis and bone cell adhesion and proliferation was shown.^54−60^ The immobilization of cyclic RGD peptide on the scaffold surface was achieved employing MP, a surface binding peptide whose sequence was inspired by DOPA-rich blue mussel foot proteins. In this study, the EC_50_ for the binding to CR was determined as 0.5 nM, which was approximately 24-fold and 228-fold lower than those of Tyr and Phe containing Bio-MP(−) and Bio-MP(--) peptides, respectively. Observed adhesion of MP to CR is in line with a number of studies showing the binding of the catechol-based coating to a wide range of surfaces including metal oxides, hydrophobic membranes, epoxy resin, and chitosan.^23,61−65^ The coating of scaffolds with MP-RGD significantly improved the cubes’ performance. In particular, as was shown by resazurin reduction and protein content (normalized to both surface area and mass) assays, the number of MG-63 and Saos-2 cells cultured for 24 h on hexagon, square, and octagon cubes having MP-RGD and fibronectin (positive control) coatings was about two times higher in comparison with that on uncoated cubes. In the same experimental setup, for the cubes with lower performance (control, hollow, and sphere inside), a positive effect of MP-RGD and/or fibronectin coatings was also detected, *i.e.*, for control, hollow, and sphere inside cubes in the protein content assay using Saos-2 cells, normalized to both surface area and mass; for hollow and control cubes in protein content assays employing MG-63 cells, normalized to both surface area and mass; for sphere inside cubes shown by resazurin reduction in Saos-2 cells; and for control, hollow, and sphere inside cubes shown by resazurin reduction in MG-63 cells. Numerous studies indicate that RGD-carrying peptides lead to an increase in cell adhesion and proliferation triggered by the binding of RGD motifs to integrin receptors expressed on cells, such as α_v_β_3_ and α_5_β_1_ expressed on bone cells.^66−68^ Indeed, following the induction of integrin clustering, large focal adhesion complexes form, enabling signal transduction within the cell and leading to cell adhesion.^69^ Also, the immobilization of ligands for RGD-specific integrin receptors may activate survival pathways preventing the execution of the apoptotic program, which is crucial at the early stage of implantation.^70^ While being cultured on scaffolds coated with RGD-containing peptides, both osteoblast-like cell lines used in this study displayed enhanced cell adhesion.^30,71^ The impact of MP-RGD, as was shown in cell culture assays and by fluorescent microscopy, was comparable to the biological activity of the fibronectin coating serving as a positive control. However, compared to the coating with the cellular expressed and large fibronectin (440 kDa), a high-purity synthetic MP-RGD peptide is more advantageous with respect to possible immune responses. Also, when being attached to the surface by mere physical adsorption, fibronectin is less stable and is prone to release resulting from the competitive protein adsorption to the material’s surface by blood serum proteins, known as the Vroman effect.^72^ Importantly, cyclic RGD-containing pentapeptides, *i.e.*, c[RGDfV], c[RGDf(*N*Me)V], and c[RGDfK], were shown to be more stable to enzymatic degradation, thus offering advantages over linear peptides.^73^ Among them, the latter, used in our research, has a high potential for biomedical applications, allowing its further functionalization or ligation employing lysine.

It should be noted that the introduction of cysteine, lysine, and propargylglycine in the mussel peptide core sequence allows the conjugation of various bioactive peptides via a thiol-maleimide Michael addition reaction, DAR_inv_, and Cu(I)-catalyzed azide–alkyne cycloaddition.^30,74^ Hence, novel multifunctional coatings ensuring the immobilization of bioactive peptides with various functions, *i.e.*, cell adhesion, wound healing, anti-inflammatory, and anticoagulant properties, synthesized as a single molecule, can be designed. Along with RGD, bioactive motifs which could be employed to generate a cooperative bioactive effect, include laminin-derived IKVAV,^75,76^ laminin-derived RNIAEIIKDI,^77^ heparin binding FHRRIKA,^74,78^ bone morphogenetic protein BMP-2-derived peptides,^79,80^ tenascin-derived VFDNFVLK,^81^ collagen-derived DGEA,^82,83^ and cadherin-derived HAV.^84^

Presently, 3D scaffolds have been designed following inspiration from various natural objects, *i.e.*, spinach leaf,^85^ the human meniscus structure,^86^ a frustule of the *Didymosphenia geminata* diatom,^87^ nacre,^88^ the dactyl club of mantis shrimp,^89^ the exoskeleton of a grasshopper,^90^ and the elytron of the figeater beetle.^91^ The scaffolds of marine glass sponges have undergone lengthy evolutionary selection in order to adapt to life at the water depth while being anchored at the ocean floor, develop damage tolerance, and establish a filtering feeding system; hence, they attract a lot of attention from the scientific community. In particular, a glass sponge *Euplectella aspergillum* was shown to possesses a square-grid architecture stabilized with a double set of diagonal bracings, which, once printed and compared with other printed lattices of the same mass, demonstrates the highest buckling resistance under a wide range of loading conditions.^92,93^ Such findings can be widely applied to the design of skyscrapers or bridges. In a study conducted by Voronkina et al.,^94^ the bioarchitecture of the siliceous honeycomb-like scaffold of *Aphrocallistes beatrix* has been described in detail. For 3D printing, the authors designed simplified models having a cylindrical shape, *i.e.*, containing flat honeycombs or tubular honeycombs with triangular openings in the walls having the potential to be employed in catalysis and bioremediation.^94^

The biomedical applicability of sponge-inspired scaffolds is supported by research aimed at the use of natural marine sponge scaffolds as ready-to-use 3D matrices for tissue engineering and regenerative medicine.^16,95,96^ In particular, human mesenchymal stem cells seeded onto chitinous scaffolds isolated from *Aplysina aerophoba* and *Aplysina fulva* sponges displayed good cell attachment and could differentiate into osteogenic lineage.^97,98^ The osteogenic tissue engineering potential of sponge skeletons was also shown for collagenous scaffolds of *Callyspongiidae* sp.^99^ and *Biemna fortis* sponges.^100^

Taken together, the use of scaffolds inspired by marine sponges open the doors for new “intelligent materials” technologies to be applied on a large scale (*i.e.*, bridges, skyscrapers, rockets, and wastewater remediation facilities) or a small scale (*i.e.*, implants, tubular organs, vascular stents, and colonic stents). The latter could also significantly benefit from the synthesis of multifunctional coatings tailored to particular applications.

## 4 Conclusions

In conclusion, (i) cloud sponge-inspired scaffolds were designed and printed from Clear Resin material, (ii) scaffolds with higher porosity (square, hexagon, octagon) displayed better biocompatibility than those with lower porosity (hollow, sphere inside) and a control cube, and (iii) the biofunctionalization of scaffolds with MP-RGD peptide enhanced their performance.

## 5 Materials and Methods

### 5.1 Scanning Electron Microscopy

The characterization of the *A. vastus* scaffold was conducted using Auriga CrossBeam (Carl Zeiss Microscopy, Jena, Germany) scanning electron microscopy at an acceleration voltage of 1 kV and a beam current of 60 pA. Images were acquired using both the InLens secondary electron detector and the energy-selected backscattered electron detector.

### 5.2 The Analysis of *A. vastus* Scaffold Morphometrical Parameters

The scanning procedure of the *A. vastus* scaffold was conducted using a Zeiss Xradia Versa 520 (Carl Zeiss Microscopy, Jena, Germany) microcomputer tomograph (micro-CT). A stack of “digital imaging and communications in medicine” DICOM images from the micro-CT were imported into the InVesalius 3.1 software package. A 3D model in STereoLithography (STL) format was generated in InVesalius 3.1 and exported into a computer-aided design (CAD) SolidWorks (Dassault Systems, USA) software package to further generate a 3D sketch. Using SolidWorks tools, a 3D sketch was converted into a solid body with its edges as cylinders with d = 0.1 mm and nodes as spheres with d = 0.5 mm. This CAD 3D model represented a replica of the *A. vastus* structure. The diameters of a sponge scaffold and of the circles inscribed in hexagons were measured employing the generated 3D model.

### 5.3 The Design and Printing of Sponge-Inspired Scaffolds

The scaffolds were designed using Inventor 2023 (California, USA) software. They were printed by a Form 2 (Formlabs, Massachusetts, USA) stereolithography printer using Clear Resin polymer (Formlabs, Massachusetts, USA). For the preprocessing, 3D printing software PreForm was applied (Formlabs, Massachusetts, USA).

### 5.4 The Synthesis of Bio-MP and MP-RGD Peptides

The synthesis of MP was conducted employing a TentaGel S RAM resin (IRIS Biotech) using standard Fmoc/*t*BU conditions.^30^ Deprotection of α-amino groups was conducted twice using 30% (v/v) piperidine (Sigma-Aldrich) in dimethylformamide (DMF, Biosolve) for 10 min. Amino acids (Fmoc-β-Ala-OH, Fmoc-Pra-OH (IRIS Biotech), Fmoc-DOPA(acetonide)-OH (Novabiochem), Fmoc-Lys(Dde)-OH, and Fmoc-Cys(Trt)-OH (IRIS Biotech)) were activated with equimolar amounts of hydroxybenzotriazole (HOBt, Novabiochem) and diisopropylcarbodiimide (DIC, IRIS Biotech) in DMF. To activate Fmoc-NH-(PEG)_2_-COOH (13 atoms, Novabiochem), equimolar amounts of 1-[bis(dimethylamino)methylene]-1*H*-1,2,3-triazolo[4,5-*b*] pyridinium 3-oxid hexafluorophosphate (HATU, Novabiochem) and *N,N*-diisopropylethylamine (DIPEA, Roth) were used. The N-terminus of MP was acetylated by incubation with acetic anhydride, DIPEA, and DMF (1:1:38) twice for 10 min. The cyclic RGD (cRGD) peptide and the Reppe anhydride lysine derivative were synthesized as described previously.^31,101^ Briefly, the peptide was elongated on 2-chlorotrityl resin (Novabiochem), generating the following sequence: Fmoc-d-Phe-Lys(Dde)-Arg(Pbf)-Gly-Asp(tBu)-resin. Following Fmoc removal, the N-terminus was protected by triphenylmethyl chloride employing DIPEA in DMF. Next, *N*-[1-(4,4-dimethyl-2,6-dioxacyclohexylidene)ethyl] (Dde) was cleaved from the Lys side chain (using 2% hydrazine in DMF), followed by the functionalization with Reppe dienophile using HOBt/DIC activation. A fully protected peptide was cleaved off the resin with glacial acetic acid, 2,2,2-trifluoroethanol, and DCM (1:1:8, v/v/v) and cyclized with HOBt/DIC in DCM for 16 h. Finally, cRGD functionalized by a Reppe dienophile was deprotected by trifluoroacetic acid (TFA) and a scavenger (see Figure S1 showing a chemical structure of c[RGDfK(Reppe)]). The functionalization of MP with cRGD has been performed using a Diels–Alder reaction with inverse electron demand (DAR_inv_).^23^ Briefly, the Dde protecting group on MP was cleaved, and the diene 5-[4-(1,2,4,5-tetrazin-3-yl)benzylamino]-5-oxopentanoic acid (Sigma-Aldrich) was coupled to the lysine side chain using 2 equiv of diene, HOBt, and DIC in DMF for 16 h (chemical structure of MP-diene is shown in Figure S1). To conduct DAR_inv_, resin loaded with diene-modified MP was swollen in water, then incubated with an aqueous solution of c[RGDfK(Reppe)] (1.5 equiv) overnight at room temperature on a shaker in an open reaction vessel (N_2_ release). After washing with water, DMF, and DCM, Fmoc was cleaved from the N-terminus. Finally, MP-RGD was cleaved from the resin and purified as described previously^23^ (chemical structure of MP-RGD is displayed in Figure S1). To test MP binding to CR, three biotin-tagged peptide derivatives were synthesized, *i.e.*, Bio-MP(+) carrying two DOPA residues and control peptides having tyrosine (Bio-MP(−)) and phenylalanine (Bio-MP(--)) residues instead (chemical structures of Bio-MP(−) and Bio-MP(--) are shown in Figure S1). Peptides were analyzed by MALDI-TOF-MS (Bruker Daltonics), and their purity was monitored by analytical RP-HPLC using a Phenomenex Jupiter4u Proteo C12 90 Å (250 mm × 4.6 mm, 4 μm, 90 Å) column and a Phenomenex Aeris Peptide 3.6u XBC18 (250 mm × 4.6 mm, 3.6 μm, 100 Å) column with a linear gradient of 10–60% (v/v) eluent B (0.08% TFA in ACN, v/v) in eluent A (0.1% TFA in water, v/v) over 40 min (Table 1, Figures S2 and S3).

**Table 1:** Analytical Data of the Synthesized Peptides^a^

peptide name	sequence	*M*_calc_ [Da]	M_found_[M + H]^+^	elution [% ACN]	purity [%]
Bio-MP(+)	Bioxx-C-EG_3_-uKu-EG_3_-K-β-NH_2_	1663.9	1664.9	23	≥95
Bio-MP(−)	Bioxx-C-EG_3_-YKY-EG_3_-K-β-NH_2_	1631.9	1633.0	25	≥95
Bio-MP(--)	Bioxx-C-EG_3_-FKF-EG_3_-K-β-NH_2_	1599.9	1600.9	30	≥95
MP-RGD	C-EG_3_-uK(diene(c[RGDfK(diehophile)]))u-EG_3_-bβ-NH_2_	2391.1	2391.9	33	≥95

a Bio = biotin; x = l-aminohexanoic acid; EG_3_ = ethylene glycole; u = l-3,4-dihydroxyphenylalanine; β = l-β-alanine; b = l-propargylglycine.

### 5.5 Peptide Binding Assay

To analyze the binding affinities of MP and its derivatives, serial dilutions (0.1, 0.5, 1, 5, 10, 50, 100, 500, and 1000 nM) of Bio-MP(+), Bio-MP(−), and Bio-MP(--) in 10 mM Tris buffer (pH 7.6) were incubated in 96-well plates containing CR discs overnight at RT while shaking. Afterward, unbound peptides were washed off using TBS-T buffer (50 mM Tris, 150 mM NaCl, 0.1% Tween20, pH 7.6), and the discs were transferred to new wells. Detection of bound peptides was established via the biotin tag in an ELISA-like assay, as described previously.^31^ In short, discs were blocked with 10% BSA in TBS buffer (50 mM Tris, 150 mM NaCl, pH 7.6), which was followed by the incubation with horseradish peroxidase-conjugated streptavidin (1:2000 in TBS containing 1% BSA). Detection was carried out using 3,3′,5,5′-tetramethylbenzidine (TMB), stopped with 1 M HCl, and quantified by reading the absorption at 450 nm (Tecan Infinite M200, Tecan Group, Männedorf, Switzerland).

### 5.6 Preparation of Samples

The samples made of CR and titanium foil (Sigma-Aldrich, USA, 0.127 mm) were sterilized in 70% ethanol in H_2_O (v/v) for 30 min while shaking and then exposed to UV light for 1 h. For coating, the samples were washed three times with sterile PBS and coated with MP-RGD (1 μM, in PBS) or fibronectin (25 μg/mL, in PBS) (used as a positive control).

### 5.7 Cell Culture

Human endothelial HMEC-1 cells were cultured in MCDB131 (Lonza, Switzerland) supplemented with 10% fetal bovine serum (FBS) (Biochrom GmbH, Germany), 10 mM glutamine (Lonza), epidermal growth factor (EGF, 10 ng/mL) (Lonza) and hydrocortisone (1 μg/mL) (Sigma, USA). Osteogenic Saos-2 cells were cultured in McCoy’s 5A (Lonza) (15% FBS, 10 mM glutamine). Another osteogenic cell line, MG-63, was cultured in EMEM (Lonza) (10% FBS and 10 mM glutamine). A suspension cell line THP-1 was cultured in RPMI (Lonza) (10% FBS and 10 mM glutamine). All cells were maintained in T75 cell culture flasks at 37 °C, 95% humidity, and 5% CO_2_ (standard conditions). These cell lines were used for no more than 15 passages. Medium was changed every 4–5 days. For cell culture assays, the following cell densities were used: 1.2 × 10^5^/mL (MG-63, Saos-2, THP-1) and 0.8 × 10^5^/mL (HMEC-1).

### 5.8 Resazurin Assay

Cell proliferation was evaluated by a resazurin conversion assay. Briefly, cells were incubated with a 1:10 volume of sterile resazurin working solution (0.025% in PBS) (Sigma-Aldrich, St. Louis, MO, USA) at 37 °C. The resulting fluorescence (excitation λ: 540 nm, emission λ: 590 nm) was measured using a Tecan Spark multimode microplate reader (Tecan Group, Männedorf, Switzerland). Blank values obtained for the respective scaffolds without cells were subtracted from the obtained fluorescence data.

### 5.9 Protein Quantification

To further investigate scaffolds’ biocompatibility, the cell density on the tested scaffolds was analyzed by quantifying the total protein content using sulforhodamine B (SRB) (SERVA Electrophoresis, Heidelberg, Germany). Briefly, without removing the cell culture medium, 100 μL of cold Fixative Reagent was added to each well. Following 1 h of incubation at 4 °C, the wells were washed four times with distilled water, and scaffolds were transferred to new wells, air-dried, and kept at room temperature until the SRB assay was performed. For 48 well cell culture plates, 400 μL of SRB was added to each well, and scaffolds were incubated for 30 min in the dark while shaking. Next, scaffolds were washed with wash solution four times and dried. Bound SRB was resolved in SRB solubilization buffer (400 μL to each well), and its absorbance was measured at 550 nm employing Tecan Infinite M200 (Tecan Group, Männedorf, Switzerland). Blank values obtained for the respective scaffolds without cells were subtracted from the obtained absorbance data.

### 5.10 Statistical Analysis

Experiments were done in triplicate. Data from *in vitro* cell culture assays were analyzed using GraphPad Prism Software version 8 (GraphPad, El Camino Real, USA) and are represented as the mean ± standard error of the mean (SEM). To compute statistical significance, One-Way Analysis of Variance (ANOVA) followed by Tukey’s posthoc test was performed. The α-level of 0.05 was set up to indicate statistical significance. In graph representations, the *p*-value is shown in the following way: *p ≤ 0.05, **p ≤ 0.01, ***p ≤ 0.001, ****p ≤ 0.0001.

## Supporting Information Available

Description of the scaffolds’ structural properties, chemical structures of peptides, identification and purification of synthesized peptides, cell viability assays of MG-63 and Saos-2 seeded on uncoated scaffolds and scaffolds coated with MP-RGD and fibronectin at 24 and 72 h, and microscopic images of MG-63 seeded on uncoated and coated (with MP-RGD and fibronectin) CR scaffolds (PDF)
