# Supplementary material for: Design and Biofunctionalization of Cloud Sponge-Inspired Scaffolds for Enhanced Bone Cell Performance
Source: ACS Appl Bio Mater. 2024 Nov 16;7(12):8281–93. doi: 10.1021/acsabm.4c01065 (PMC11653246; doi:10.1021/acsabm.4c01065)
Supplement: Supplementary file 1 — mt4c01065_si_001.pdf [file mt4c01065_si_001.pdf]

Supporting Information

Design and biofunctionalization of cloud-sponge inspired scaffolds for enhanced bone cell performance

Philipp Zimmermann[1], Peter Schulze[1], Annette G. Beck-Sickinger[2], Yuliya Khrunyk[2*]

[1]Leipzig University of Applied Sciences (HTWK), Engineering Faculty, Karl Liebknecht Str. 134, D-04277, Leipzig, Germany

[2]Institute of Biochemistry, Faculty of Life Sciences, Leipzig University, Brüderstrs. 34, D-04103, Leipzig, Germany

* yulyia.khrunyk@uni-leipzig.de; Tel.: +49 1792271413

Table S1. The description of scaffolds´ structural properties.

	hexagon	*square*	*octagon*	*hollow*	*sphere inside*	*control*
**dimensions (xyz), mm**	**7x7x6**	**7x7x6**	**7x7x6**	**7x7x6**	**7x7x6**	**7x7x6**
**main structural element, mm**	**0.8**	**0.8**	**0.8**	**0.8**	**0.9**	**-**
**string thickness, mm**	**0.1**	**0.1**	**0.1**	**0.1**	**-**	**-**
**surface area (CAD), mm^2**	**1,425.780**	**416.960**	**659.766**	**1,321.591**	**1,080.850**	**266.000**
**volume (CAD), mm^3**	**42.191**	**10.874**	**18.440**	**63.164**	**83.704**	**294.000**
**porosity, %**	**86**	**96**	**94**	**79**	**72**	**-**

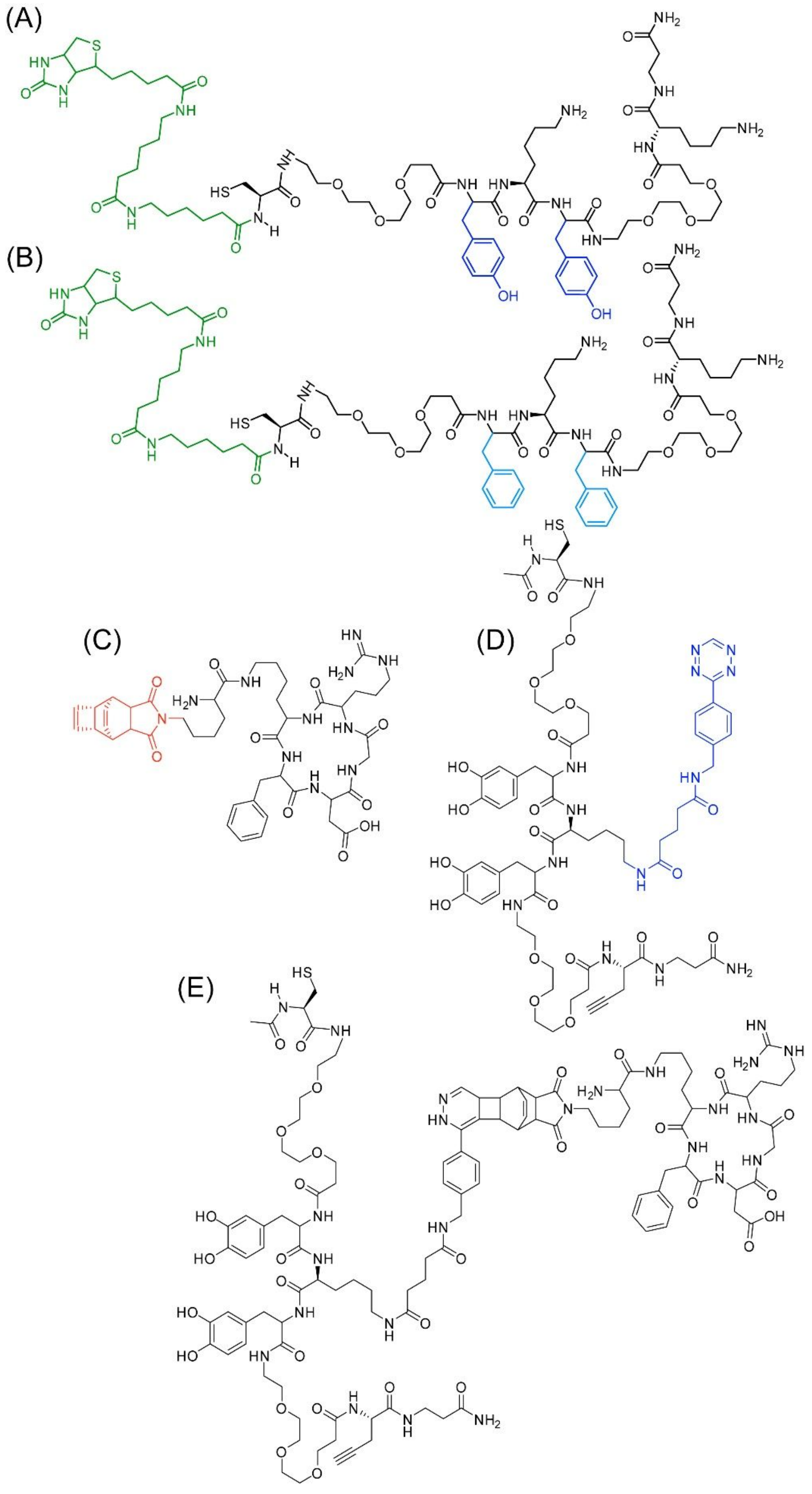

Figure S1. Chemical structures of peptides: (A) Bio-MP(-) and (B) Bio-MP(--) with DOPA being replaced by Tyr (blue color) and Phe (light blue), respectively. Both Bio-MP(-) and Bio-MP(--) are equipped with aminohexanoic acid spacers and a biotin tag (green color). (C) c[RGDfK(Reppe)], Reppe dienophile is shown in red; (D) MP-diene (diene is shown in blue); (E) MP-RGD.

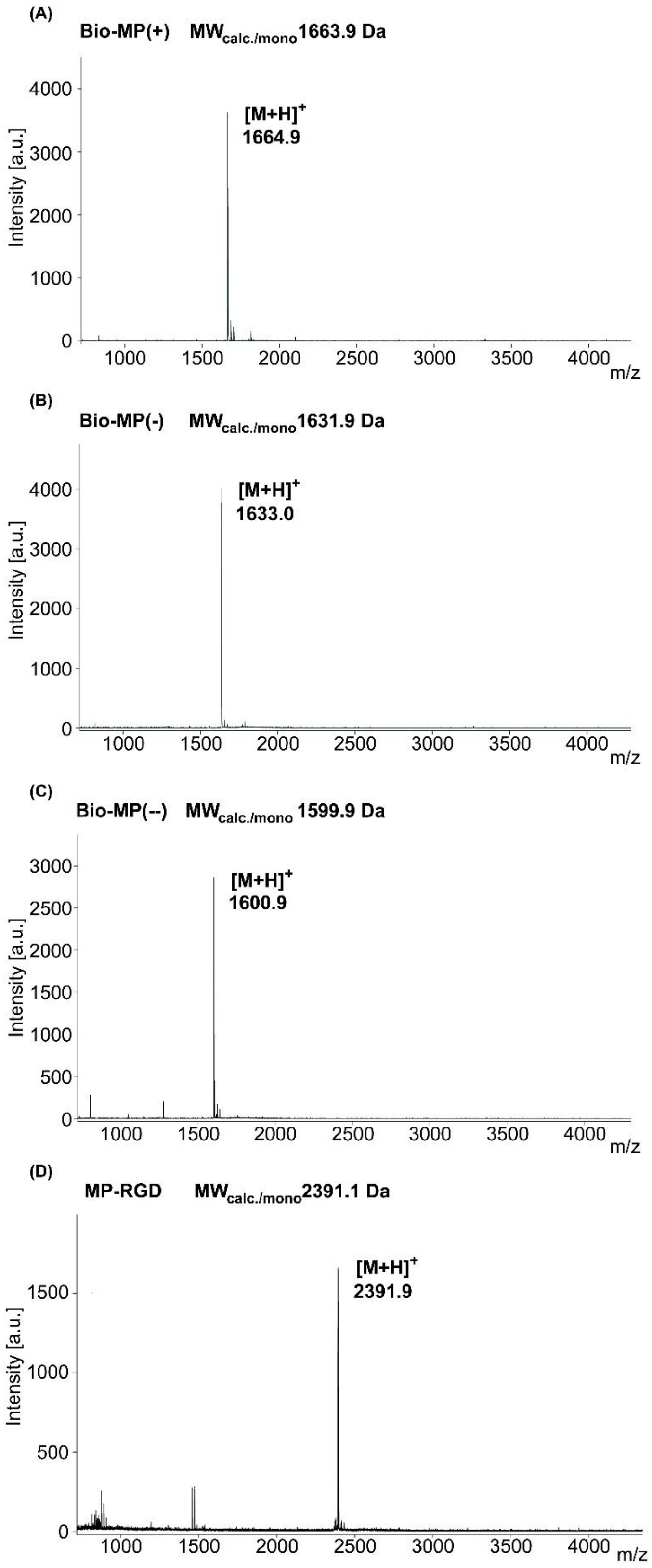

Figure S2. MALDI-ToF-MS of purified peptides Bio-MP(+) (A), Bio-MP(-) (B), Bio-MP(--) (C), and MP-RGD (D).

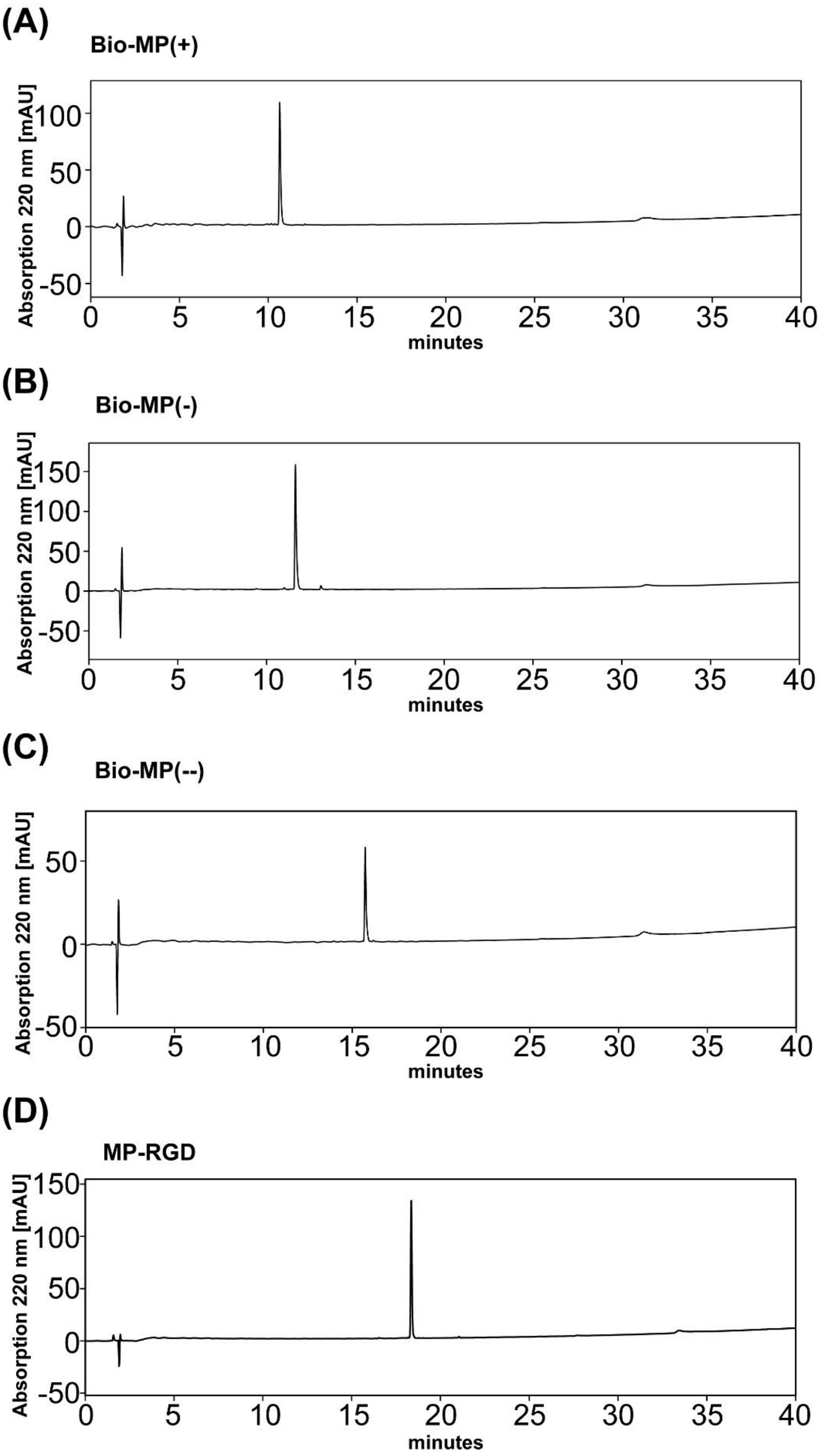

Figure S3. RP-HPLC of purified peptides Bio-MP(+) (A), Bio-MP(-) (B), Bio-MP(--) (C), and MP-RGD (D) using a Phenomenex Aeris® Peptide 3.6u XBC18 (250 mm×4.6 mm, 3.6 µm, 100 Å) column. Chromatograms were recorded with a linear gradient of 10-60% (v/v) eluent B (0.08% TFA in ACN, v/v) in eluent A (0.1% TFA in water, v/v) over 40 min.

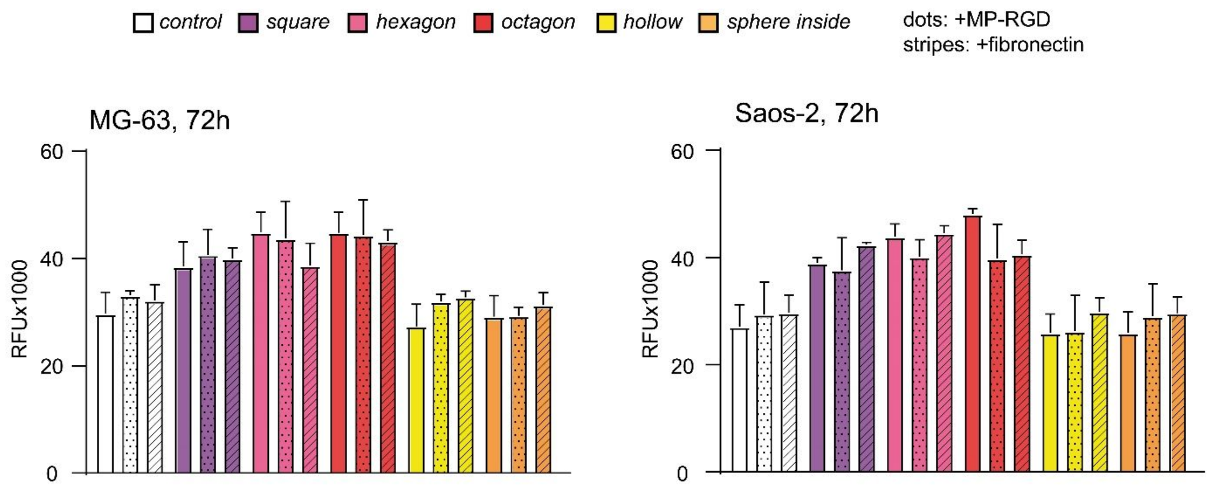

Figure S4. Resazurin reduction in MG-63 and Saos-2 cell cultures at 72 h after seeding, indicating mitochondrial activity. Cells were cultured on uncoated scaffolds and coated scaffolds (MP-RGD, 1 µM, dots; fibronectin, 25 µg/ml, stripes). The measurements were taken at 120 min following incubation with resazurin; RFU: relative fluorescence units; data represent mean ± SEM, n=3.

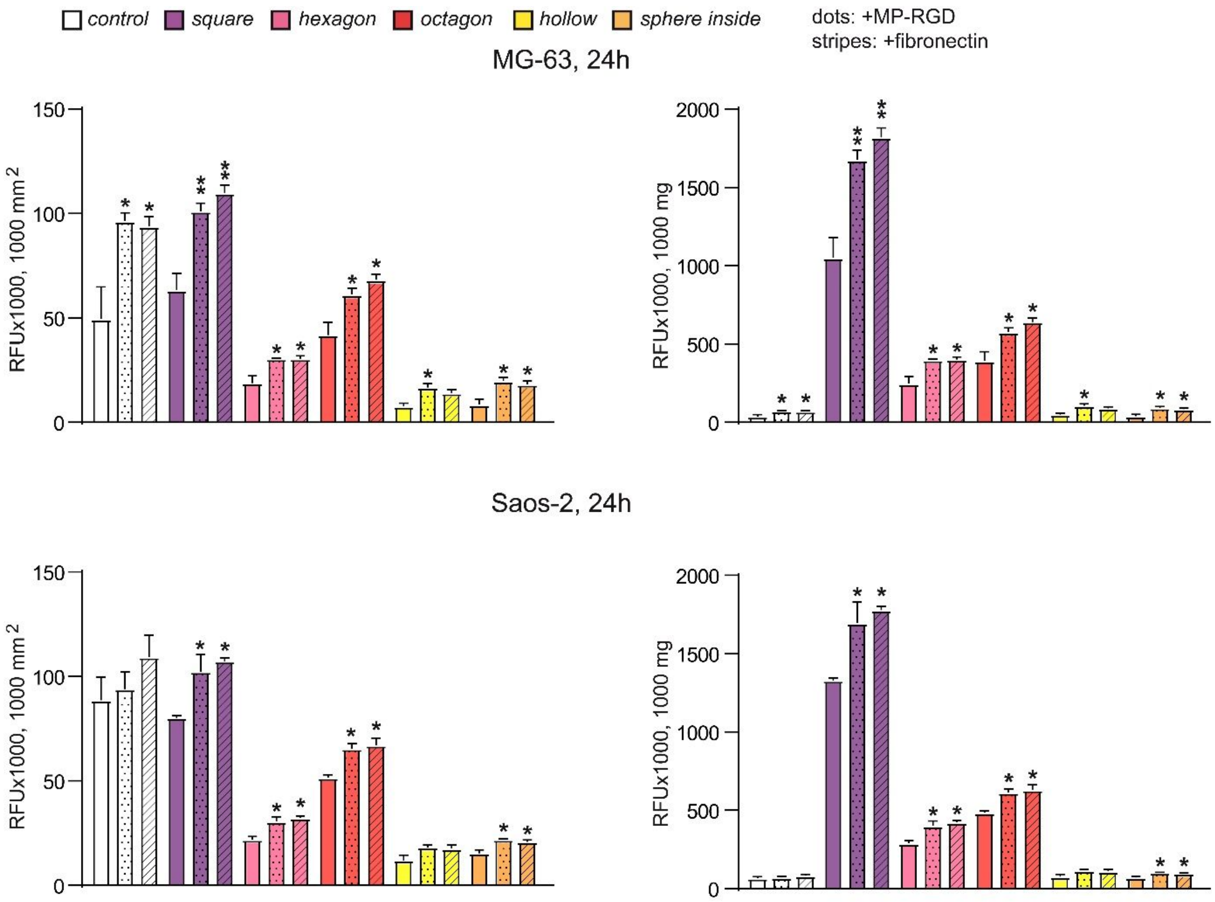

Figure S5. Resazurin reduction in MG-63 and Saos-2 cell cultures at 24 h after seeding, indicating mitochondrial activity. Cells were cultured on uncoated scaffolds and coated scaffolds (MP-RGD, 1 µM, dots; fibronectin, 25 µg/ml, stripes). The measurements were taken at 120 min following incubation with resazurin; RFU: relative fluorescence units; the data are normalized to the scaffolds´ surface area (1000 mm^2) and mass (1000 mg); * for $p \leq 0.05$, ** for $p \leq 0.01$, significance is shown in comparison to uncoated scaffolds; data represent mean ± SEM, n=3.

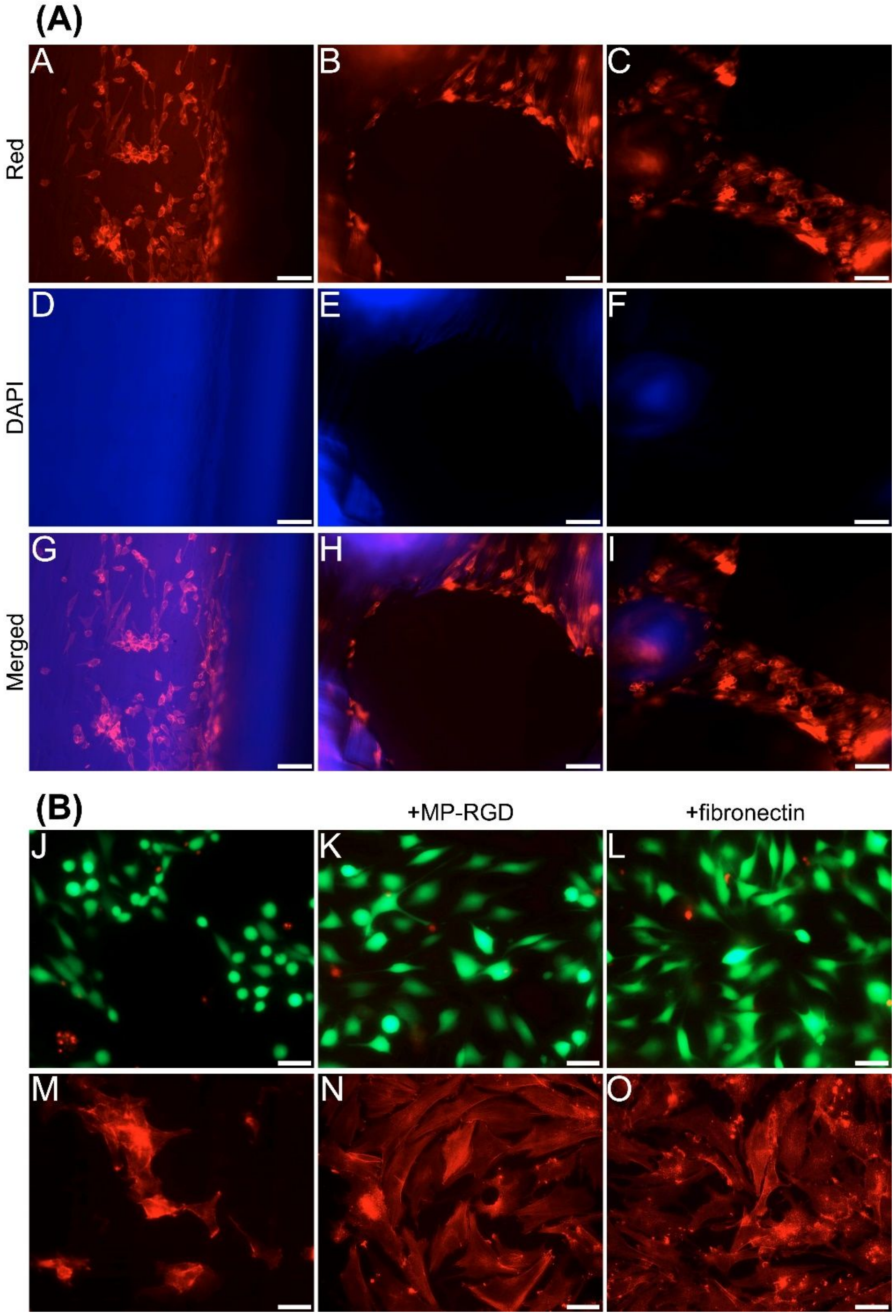

Figure S6. Microscopic images of MG-63 cells 24h post seeding onto (A) CR control cube scaffolds ((A): A, D, G), porous scaffolds, i.e. octagon ((A): B, E, H) and square ((A): C, F, I), and on (B) CR discs uncoated ((B): J, M), MP-RGD-coated (1 µM; (B): K, N) and fibronectin-coated (25 µg/ml; (B): L, O). Cell actin was stained by TRITC-Phalloidin (red) ((A), (B): M-O). Nuclei cannot be visible on CR material due to its autofluorescence in DAPI channel (autofluorescent images of scaffolds are shown in (A): D-I (DAPI; merged). Viable cells were visualized in (B): J-L: prior to microscopy, cells were treated with Calcein-AM / Propidium Iodide Double Staining Kit (Sigma-Aldrich) allowing simultaneous fluorescence staining of viable (green fluorescence) and dead (red fluorescence) cells. Sb=100µm in (A) and 50µm in (B). Both coated scaffolds (by MP-RGD and fibronectin) were characterized by a well-spread cell pattern, while untreated scaffolds showed a poorer cell attachment.

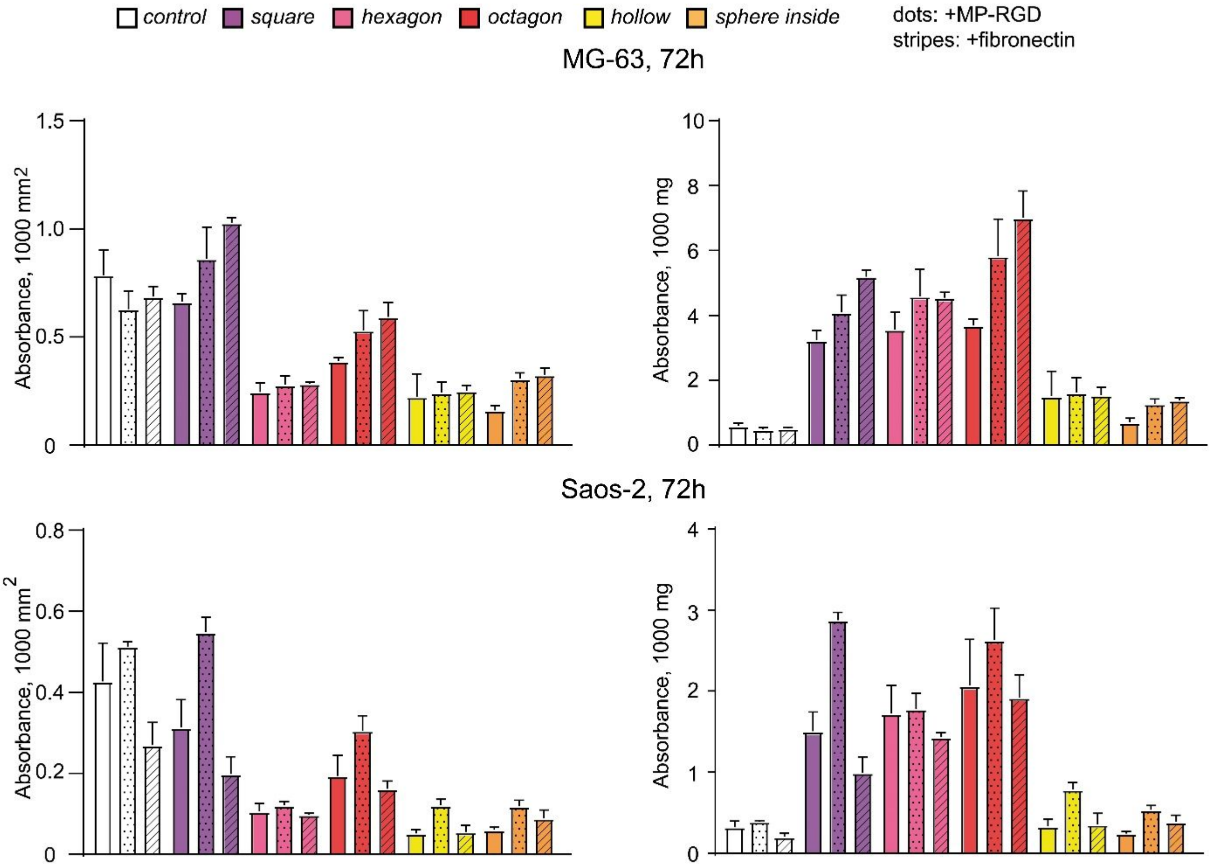

Figure S7. Sulforhodamine B (SRB) assay detecting scaffolds biocompatibility based on cellular protein content. SRB absorbance is shown for tested scaffolds seeded with MG-63 and Saos-2 cells, 72h cell culture. Cells were cultured on uncoated scaffolds and coated scaffolds (MP-RGD, 1 μM, dots; fibronectin, 25 μg/ml, stripes). The data are normalized to the scaffolds´ surface area (1000 mm^2) and mass (1000 mg); data represent mean ± SEM, n=3.
